# Supplementary material for: A novel criteria on exponentially passive analysis for Takagi-Sugeno fuzzy of neutral dynamic system with various time-varying delays
Source: PLoS One. 2022 Oct 7;17(10):e0275057. doi: 10.1371/journal.pone.0275057 (PMC9544024; doi:10.1371/journal.pone.0275057)
Supplement: Attachment — Submitted filename: Original Manuscript ID round 3.pdf [file pone.0275057.s005.pdf]

Original Manuscript ID: PONE-D-22-00021R2

Original Article Title: “A novel criteria on exponentially passive analysis for Takagi-Sugeno fuzzy of neutral dynamic system with various time-varying delays”

To: PLOS ONE Editor

Re: Response to reviewers

Dear Editor,

Thank you for allowing a resubmission of our manuscript, with an opportunity to address the reviewers’ comments.

We are uploading (a) our point-by-point response to the comments (below) (response to reviewers), (b) an updated manuscript with yellow highlighting indicating changes, and (c) a clean updated manuscript without highlights (PDF main document).

Best regards,

<Thongchai Botmart> et al.

Journal Requirements: Please review your reference list to ensure that it is complete and correct. If you have cited papers that have been retracted, please include the rationale for doing so in the manuscript text, or remove these references and replace them with relevant current references. Any changes to the reference list should be mentioned in the rebuttal letter that accompanies your revised manuscript. If you need to cite a retracted article, indicate the article's retracted status in the References list and also include a citation and full reference for the retraction notice.

Answer: We have added the referent [40] because a reviewer requires us to compare the difference between the work in the following paper [New results on robust exponential stability of Takagi–Sugeno fuzzy for neutral differential systems with mixed time-varying delays] and the work in this paper. I.e., New results on robust exponential stability of Takagi - Sugeno fuzzy for neutral differential systems with mixed time-varying delays [40] only focus on exponential stability for neutral differential equations of the uncertain Takagi - Sugeno fuzzy system. This paper studies exponential stable and exponentially passive neutral differential equations of the uncertain Takagi - Sugeno fuzzy system for further improvement. Furthermore, we consider mixed interval time-varying delays: mixed interval discrete time-varying delay, interval distributed time-varying delay, and interval neutral time-varying delay, i.e., $\sigma(t)$ is interval discrete time-varying delay which satisfies $0 \leq \sigma_1 \leq \sigma(t) \leq \sigma_2$, $h(t)$ is interval distributed time-varying delays which satisfy $0 \leq h_1 \leq h(t) \leq h_2$. And $\tau(t)$ is an interval neutral time-varying delay that satisfies $0 \leq \tau_1 \leq \tau(t) \leq \tau_2$.

[40] Tranthi J, Botmart T, Weera W, La-inchua T, Pinjai S. New results on robust exponential stability of Takagi - Sugeno fuzzy for neutral differential systems with mixed time-varying delays. Math Comput Simul. 2022; 201 : 714-738.

Review Comments to the Author

Reviewer #2: In response to the reviewers' comments, the authors improved their revised manuscript, and now it can be published.

Answer: we greatly appreciate your kind comments and acceptance for our manuscript.

Reviewer #3: The authors have improved the revised manuscript based on the reviewers' comments, All my comments have been addressed—no further comments.

Answer: we greatly appreciate your kind comments and acceptance for our manuscript.
