# Supplementary material for: A novel criteria on exponentially passive analysis for Takagi-Sugeno fuzzy of neutral dynamic system with various time-varying delays
Source: PLoS One. 2022 Oct 7;17(10):e0275057. doi: 10.1371/journal.pone.0275057 (PMC9544024; doi:10.1371/journal.pone.0275057)
Supplement: Attachment — Submitted filename: review.docx [file pone.0275057.s001.docx]

Review

Title : A novel criteria on exponentially passive analysis for Takagi-Sugeno

fuzzy of neutral dynamic system with various time-varying delays

This study is relevant to designing reliable exponentially passive analysis for T-S fuzzy of dynamic systems with various time-varying delays such as neutral, discrete, and distributed time-varying delays. By using the Lyapunov-Krasovskii function, The Newton–Leibniz theory, the zero equations, and the matrix inequality technique. The multiple delay-dependent criterion with assure exponential stability and passive performance on the discussed T-S fuzzy system, are defined in respect of linear matrix inequalities (LMIs) that can be checked easily using LMI toolbox of MATLAB. A less conservative exponentially passive criterion given for special cases of general stability of a neutral differential system, by those approaches. The results of this study are delay-dependent, which depend on the lower bound and the upper bound with the time-varying delay. Lastly, the examples of Numerical are presented the performance of our criteria with the results obtained summarize some of the previous results as well.

I think the readers of this journal will appreciate the results of this manuscript. Generally speaking, the manuscript is well written, the material is judiciously divided and organized and correct from scientific point of view. For these reasons I can recommend the acceptance of this paper after some corrections presented below:

1. It seems that the proof of theorem 1 is so complicated, and for such many complex and large-scale matrices, how to guarantee the existence of the LMI problem for eq. (2).
2. The novelty of this paper should be highlighted in the main contributions through comparing it with the existing works.
3. The linguist should be thoroughly examined and corrected. Numerous typos and grammatical errors appear in the manuscript. For example,

3.4 On page 11, line 14, $"-D\int_{t-h(t)}^{t} x\left( s \right)ds- \left. \left. Eu\left( t \right) \right) \right]0"$ should be $"-D\int_{t-h(t)}^{t} x\left( s \right)ds- \left. \left. Eu\left( t \right) \right) \right]"$.

3.5 On page 19, line 143, “since lemma 5” should be “Since lemma 5”.

3.6 On page 29, line 181, $"D+\Delta D_{i}\left( t \right)=0$ and $E+\Delta E_{i}\left( t \right)=0"$ should be $"D_{i}+\Delta D_{i}\left( t \right)=0$ and $E_{i}+\Delta E_{i}\left( t \right)=0"$.

3.7. On page 33, line 214, $"C+\Delta C_{i}\left( t \right)=0, D+\Delta D_{i}\left( t \right)=0$ and $E+\Delta E_{i}\left( t \right)=0"$ should be $"C_{i}+\Delta C_{i}\left( t \right)=0, D_{i}+\Delta D_{i}\left( t \right)=0$ and $E_{i}+\Delta E_{i}\left( t \right)=0"$.

4. What is “interval discrete time-varying delay”? What are the differences between it and time-varying delay? Besides, what is “mixed interval time-varying delays”?
5. The main conclusions of this paper are given in the form of LMI, while, the LMI derived in this paper contains so many information of the system, which is very difficult to verify, so some simple method should be considered, i.e., a simple Lyapunov-Krasovskii functional should be considered.
6. Based on the obtained results, some potential improvements or future work could be discussed in the Conclusion.
