# Supplementary material for: A novel criteria on exponentially passive analysis for Takagi-Sugeno fuzzy of neutral dynamic system with various time-varying delays
Source: PLoS One. 2022 Oct 7;17(10):e0275057. doi: 10.1371/journal.pone.0275057 (PMC9544024; doi:10.1371/journal.pone.0275057)
Supplement: Attachment — Submitted filename: PLoS-ONE-Response-to-Reviewers pang.docx [file pone.0275057.s002.docx]

**Original Manuscript ID: PONE-D-22-00021**

**Original Article Title: “A novel criteria on exponentially passive analysis for Takagi-Sugeno fuzzy of neutral dynamic system with various time-varying delays”**

To: PLOS ONE Editor

Re: Response to reviewers

Dear Editor,

Thank you for allowing a resubmission of our manuscript, with an opportunity to address the reviewers’ comments.

We are uploading (a) our point-by-point response to the comments (below) (response to reviewers), (b) an updated manuscript with yellow highlighting indicating changes, and (c) a clean updated manuscript without highlights (PDF main document).

Best regards,

<Thongchai Botmart> et al.

**Reviewer #1**

This study is relevant to designing reliable exponentially passive analysis for T-S fuzzy of dynamic systems with various time-varying delays such as neutral, discrete, and distributed time-varying delays. By using the Lyapunov-Krasovskii function, The Newton–Leibniz theory, the zero equations, and the matrix inequality technique. The multiple delay-dependent criterion with assure exponential stability and passive performance on the discussed T-S fuzzy system, are defined in respect of linear matrix inequalities (LMIs) that can be checked easily using LMI toolbox of MATLAB. A less conservative exponentially passive criterion given for special cases of general stability of a neutral differential system, by those approaches. The results of this study are delay-dependent, which depend on the lower bound and the upper bound with the time-varying delay. Lastly, the examples of Numerical are presented the performance of our criteria with the results obtained summarize some of the previous results as well.

I think the readers of this journal will appreciate the results of this manuscript. Generally speaking, the manuscript is well written, the material is judiciously divided and organized and correct from scientific point of view. For these reasons I can recommend the acceptance of this paper after some corrections presented below:

**Answer :** We really appreciate your suggestion. We have revised all of them as the follows:

1. It seems that the proof of theorem 1 is so complicated, and for such many complex and large-scale matrices, how to guarantee the existence of the LMI problem for eq. (2).

**Author response:** The stability criterion of Theorem 1 in the form LMIs (2) can be easily to examine by using LMI toolbox in MATLAB. The improved stability conditions by constructing new Lyapunov functionals is based on LMIs and the dimension of the LMIs depends on the number of premise variables of fuzzy set . Thus, the computational burden problem goes up. This problem is the issue in studying needs of LMI optimization in applied mathematics and the optimization research. Hence, in the further, new techniques to reduce the conservativeness caused by the time-delays such as the delay-fractioning approach and so on. Moreover, the vectors can be solve directly solution of LMI approach. Moreover, the effectiveness of computer should update the performance computer which can be helpful a computational problem.

**Author action: -**

2. The novelty of this paper should be highlighted in the main contributions through comparing it with the existing works.

**Author response:** We have already rewritten by added the main contribution of our paper.

**Author action:** We updated the manuscript in paragraph 5 of the introduction by adding the main contribution of our paper as follows:

• We construct the suitable Lyapunov-Krasovskii functional, which consists of single, double, triple, and quadruple integral terms containing information about the lower and upper bounds of the delays $\sigma_{2}, \tau_{2}$ and a state $x\left( t \right)$. Furthermore, the LKF contains a new triple integral term

$$\sigma_{2}^{2}\int_{-\sigma_{2}}^{0} \int_{\theta}^{0} \int_{t+\beta}^{t} e^{2\alpha\left( s-t \right)}\dot{x}^{T}(s)W_{1}\dot{x}(s)dsd\beta d\theta, \tau_{2}^{2}\int_{-\tau_{2}}^{0} \int_{\theta}^{0} \int_{t+\beta}^{t} e^{2\alpha\left( s-t \right)}\dot{x}^{T}(s)W_{2}\dot{x}(s)dsd\beta d\theta$$

and a new quadruple integral term

$$\sigma_{2}^{3}\int_{-\sigma_{2}}^{0} \int_{v}^{0} \int_{\theta}^{0} \int_{t+\beta}^{t} e^{2\alpha\left( s-t \right)}\dot{x}^{T}(s)U_{1}\dot{x}(s)dsd\beta d\theta dv, \tau_{2}^{3}\int_{-\tau_{2}}^{0} \int_{v}^{0} \int_{\theta}^{0} \int_{t+\beta}^{t} e^{2\alpha\left( s-t \right)}\dot{x}^{T}(s)U_{2}\dot{x}(s)dsd\beta d\theta dv$$

that do not appear in [7, 25-29].

• For the first time, an improved Wirtinger inequality, a new triple integral inequality, and zero equation together with convex combination approach are used in this work; as a result, we obtain more general results and maximum allowable delay bounds greater than in previous literature [7, 25-29].

3. The linguist should be thoroughly examined and corrected. Numerous typos and grammatical errors appear in the manuscript. For example,

3.1 On page 11, line 14, $"-D\int_{t-h(t)}^{t} x\left( s \right)ds- \left. \left. Eu\left( t \right) \right) \right]0"$ should be $"-D\int_{t-h(t)}^{t} x\left( s \right)ds- \left. \left. Eu\left( t \right) \right) \right]"$.

3.2 On page 19, line 143, “since lemma 5” should be “Since lemma 5”.

3.3 On page 29, line 181, $"D+\Delta D_{i}\left( t \right)=0$ and $E+\Delta E_{i}\left( t \right)=0"$ should be $"D_{i}+\Delta D_{i}\left( t \right)=0$ and $E_{i}+\Delta E_{i}\left( t \right)=0"$.

3.4. On page 33, line 214, $"C+\Delta C_{i}\left( t \right)=0, D+\Delta D_{i}\left( t \right)=0$ and $E+\Delta E_{i}\left( t \right)=0"$ should be $"C_{i}+\Delta C_{i}\left( t \right)=0, D_{i}+\Delta D_{i}\left( t \right)=0$ and $E_{i}+\Delta E_{i}\left( t \right)=0"$.

**Author response:** Thanks for the suggestion we have already and carefully rechecked and revised the entire paper.

**Author action:** We have corrected the manuscript by rewriting it as follows:

1. On page 1, line 4, “exponential stability and passive performance” should be “exponentially passive”.

2. On page 3, line 105, “exponential stability and passivity criteria” should be “exponentially passive criteria”.

3. On page 3, line 107, “exponential stability and passive performance” should be “exponentially passive”.

4. On page 3, line 109, “the general phase value system of the neutral differential system” should be “the general phase value system for the neutral dynamic system”.

5. On page 3, line 112, “the neutral differential system” should be “the neutral dynamic system”.

6. On page 11, line 14, $"-D\int_{t-h(t)}^{t} x\left( s \right)ds- \left. \left. Eu\left( t \right) \right) \right]0"$ should be $"-D\int_{t-h(t)}^{t} x\left( s \right)ds- \left. \left. Eu\left( t \right) \right) \right]"$.

7. On page 12, line 8, $"\left[ \left[ x\left( t \right)" \right. \right.$ should be $"\left[ x(t) \right."$.

8. On page 12, line 9, $"\left[ \left[ x\left( t \right)" \right. \right.$ should be $"\left[ x(t) \right."$.

9. On page 12, line 16, $"\left[ \left[ x\left( t \right)" \right. \right.$ should be $"\left[ x(t) \right."$.

10. On page 12, line 17, $"\left[ \left[ x\left( t \right)" \right. \right.$ should be $"\left[ x(t) \right."$.

11. On page 12, line 21, $"\left[ \left[ x\left( t \right)" \right. \right.$ should be $"\left[ x(t) \right."$.

12. On page 12, line 22, $"\left[ \left[ x\left( t \right)" \right. \right.$ should be $"\left[ x(t) \right."$.

13. On page 13, line 7, $"\left[ \left[ x\left( t \right)" \right. \right.$ should be $"\left[ x(t) \right."$.

14. On page 13, line 8, $"\left[ \left[ x\left( t \right)" \right. \right.$ should be $"\left[ x(t) \right."$.

15. On page 19, line 143, “since lemma 5” should be “Since lemma 5”.

16. On page 29, line 181, $"D+\Delta D_{i}\left( t \right)=0$ and $E+\Delta E_{i}\left( t \right)=0"$ should be $"D_{i}+\Delta D_{i}\left( t \right)=0$ and $E_{i}+\Delta E_{i}\left( t \right)=0"$.

17. On page 30, line 194, “exponential stability criterion and passivity performance” should be “exponentially passive”.

18. On page 33, line 214, $"C+\Delta C_{i}\left( t \right)=0, D+\Delta D_{i}\left( t \right)=0$ and $E+\Delta E_{i}\left( t \right)=0"$ should be $"C_{i}+\Delta C_{i}\left( t \right)=0, D_{i}+\Delta D_{i}\left( t \right)=0$ and $E_{i}+\Delta E_{i}\left( t \right)=0"$.

4. What is “interval discrete time-varying delay”? What are the differences between it and time-varying delay? Besides, what is “mixed interval time-varying delays”?

**Author response:**

-Interval discrete time-varying delay is interval time-varying delay and the lower bound is not restricted to zero which is the state variable.

-Time-varying delay is the delay which depends on time such as state delay, distributed delay, or interval time-varying delay.

-In this work, mixed interval time-varying delays are the delays which are mixed interval discrete time-varying delay, interval distributed time-varying delay and interval neutral time-varying delay i.e., $\sigma(t)$is interval discrete time-varying delay which satisfies $0\leq\sigma_{1}\leq\sigma(t)\leq\sigma_{2}$, $h(t)$ is interval distributed time-varying delays which satisfy $0\leq h_{1}\leq h\left( t \right)\leq h_{2}.$ and $\tau(t)$ is interval neutral time-varying delay which satisfy $0\leq\tau_{1}\leq\tau(t)\leq\tau_{2}$.

**Author action: -**

5. The main conclusions of this paper are given in the form of LMI, while, the LMI derived in this paper contains so many information of the system, which is very difficult to verify, so some simple method should be considered, i.e., a simple Lyapunov-Krasovskii functional should be considered.

**Author response:** In this work, the Lyapunov–Krasovskii functional contains single, double, triple, and quadruple integral terms, in which full information on the delays and a state variable is used. Therefore, the construction and the technique for computation of the Lyapunov–Krasovskii functional are the main keys to improve results of this work. In the proof of Theorems 1 improved Wirtinger’s single integral inequality, a novel triple integral inequality, and convex combination technique are used to bound the derivative of Lyapunov– Krasovskii functional, which provide tighter bound than the inequalities. All of these lead to a reduction of the conservatism of our results compared to those in some exiting works and, in particular, numerical examples. However, the complex computation of the Lyapunov–Krasovskii functional leads to the LMI derived in this work which contains much information about the system. Hence, for further work, it is interesting for researchers to improve the technique for a simple Lyapunov–Krasovskii functional and also achieve better results

**Author action: -**

6. Based on the obtained results, some potential improvements or future work could be discussed in the Conclusion.

**Author response:** We have already rewritten in paragraph 3 of the introduction and the conclusion of our paper.

**Author action:** We have updated the manuscript by rewritten in paragraph 3 of the introduction and the conclusion. In addition, we added the six references as follows:

“**In paragraph 3 of the introduction**

In addition, passivity theory is another proficient tool for analyzing system stability. The passive theory is the main pointed to the system can keep the systems internal stability which is the passive properties. So that, the problem of inactivity is therefore an important part of recent research. Then, the passive control uses the product of output and input as the power rating, which captures the attenuation properties of the system under the bounded input. In particular, passivity theory is more general than stability theory because it can be illustrated Lyapunov function under the theory of stabilization. This theory is used for issue of engineering i.e. electric circuits and heat energy systems. Nowadays many researchers have studied passive theory and passive control problems extensively, for instance, Zhang et al. [11] who studied the passive controller design issue with both state and input delays for a class of continuous-time T-S fuzzy systems. Another researcher such as Wu et al. [12] identified the problem of passive control for fuzzy network systems, considering the random uncertainty variable sampling interval and the delay caused by the fixed network. Similarly, Song and He [13] who researched the robust passive control is offered for a limited time for nonlinear systems with time-delay. The studied of Yu et al. [14] focused both passive analysis and passive control for erratic intermittent switching delay systems through a simple switching signal design. Likewise, Yotha et al. Improved delay-dependent approach to passivity analysis for uncertain neural networks with discrete interval and distributed time-varying delays [15]. So, it is challenging to solve exponentially passive for T-S fuzzy of neutral dynamic system with various time-varying delays.”

**“Conclusion**

This study rectify the exponentially passive analysis for the neutral difference of the uncertain Takagi-Sukeno fuzzy system with neutral, discrete and distributed interval time-varying delayed. By spending the Newton-Leibniz formulas, Lyapunov-Krasovskii functions (LKF), zero equations and matrix inequality techniques. The form of linear matrix inequalities (LMIs) is constructed from the exponentially passive which the numerical efficiency can be verified. Hence, this study shows the example of numbers to demonstrate the effectiveness of our theoretical results, and to illustrate, the results we have obtained are less conservative than the results available in other works. In the future work, the derived results and methods in this paper are expected to be applied to other systems such as fuzzy generalized complex-valued, guaranteed cost, pinning control of neural networks for impulsive effects on stability and passivity analysis, and so on [40–44].”

**Reviewer #2:** In this study, the authors investigate an exponentially passive analysis for T-S fuzzy dynamical systems with various time-varying delays, including neutral, discrete, and distributed time-varying delays using the Lyapunov-Krasovskii function, Newton-Leibniz theory, zero equations, and matrix inequality. As compared to literature, they obtained less conservative results.

The theoretical analysis appears to be correct, but the paper must undergo careful and critical editing of both the English language and English grammar. There are many linguistic omissions, errors, and oversights throughout. There are several such errors scattered throughout the

manuscript, so it is very important to check it carefully**.**

**Answer :** We really appreciate your suggestion. We have checked the grammatical problems throughout the manuscript thoroughly already.
