# Supplementary material for: A novel criteria on exponentially passive analysis for Takagi-Sugeno fuzzy of neutral dynamic system with various time-varying delays
Source: PLoS One. 2022 Oct 7;17(10):e0275057. doi: 10.1371/journal.pone.0275057 (PMC9544024; doi:10.1371/journal.pone.0275057)
Supplement: Attachment — Submitted filename: PLoS-ONE-Response-to-Reviewers.docx [file pone.0275057.s004.docx]

Original Manuscript ID: PONE-D-22-00021R1

Original Article Title: “A novel criteria on exponentially passive analysis for Takagi-Sugeno fuzzy of neutral dynamic system with various time-varying delays”

To: PLOS ONE Editor

Re: Response to reviewers

Dear Editor,

Thank you for allowing a resubmission of our manuscript, with an opportunity to address the reviewers’ comments.

We are uploading (a) our point-by-point response to the comments (below) (response to reviewers), (b) an updated manuscript with yellow highlighting indicating changes, and (c) a clean updated manuscript without highlights (PDF main document).

Best regards,

<Thongchai Botmart> et al.

**Reviewer #2:** The authors have improved the revised manuscript based on the reviewers' comments, but some additional corrections are needed. Please see the attached file.

**Answer :** We appreciate your suggestion. We revised as the suggestion already and the English of the whole paper is carefully checked in the revised paper.

**Reviewer #3:**

1. The abstract needs to be written in a more clarified way indicating the contribution.

**Author response:** We have already rewritten the abstract.

**Author action:** We updated the manuscript in the abstract part as follows:

This paper is the first studying on designing exponentially passive analysis for T-S fuzzy of dynamic systems with various time-varying delays such as neutral, discrete, and distributed time-varying delays. Constructing the new Lyapunov-Krasovskii function and the Newton-Leibniz theory, the zero equations, and the matrix inequality techniques, the multiple delay-dependent criteria, with assuring exponentially passive on the discussed T-S fuzzy system, are defined in respect of linear matrix inequalities (LMIs) that can be checked easily using the LMI toolbox of MATLAB. Those approaches give less conservative, exponentially passive criteria for special cases of general stability of a neutral differential system. Furthermore, the results of this study are delay-dependent, which depend on the lower and upper bound with the time-varying delay. Lastly, some numerical examples illustrate the performance of our criteria based on the results obtained and summarize some of the previous achievements.

2. The authors have to state the weakness in previous works which motivated this study.

**Author response:** Many thanks for your valuable remarks.

**Author action:** We have revised in introduction part and added a Remark 1. as follows the above mentioned, there is still room for further improvement: the fuzzy T-S method with delay-dependent based on latency to the possible extent of the thresholds for exponential stability and passivity performance.

**Remark 1** This study constructs the suitable Lyapunov-Krasovskii functional, which consists of single, double, triple, and quadruple integral terms containing information about the lower and upper bounds of the delays $\sigma_{2}, \tau_{2}$ and a state $x\left( t \right)$. Furthermore, the LKF contains a new triple integral term as follows:

$$\sigma_{2}^{2}\int_{-\sigma_{2}}^{0} \int_{\theta}^{0} \int_{t+\beta}^{t} e^{2\alpha\left( s-t \right)}\dot{x}^{T}(s)W_{1}\dot{x}(s)dsd\beta d\theta, \tau_{2}^{2}\int_{-\tau_{2}}^{0} \int_{\theta}^{0} \int_{t+\beta}^{t} e^{2\alpha\left( s-t \right)}\dot{x}^{T}(s)W_{2}\dot{x}(s)dsd\beta d\theta$$

and a new quadruple integral term

$$\sigma_{2}^{3}\int_{-\sigma_{2}}^{0} \int_{v}^{0} \int_{\theta}^{0} \int_{t+\beta}^{t} e^{2\alpha\left( s-t \right)}\dot{x}^{T}(s)U_{1}\dot{x}(s)dsd\beta d\theta dv, \tau_{2}^{3}\int_{-\tau_{2}}^{0} \int_{v}^{0} \int_{\theta}^{0} \int_{t+\beta}^{t} e^{2\alpha\left( s-t \right)}\dot{x}^{T}(s)U_{2}\dot{x}(s)dsd\beta d\theta dv$$

that do not appear in [7, 25-29]. These improvement techniques enhance to get better results.

3. The authors did not explain the difference between the work in the following paper [New results on robust exponential stability of Takagi–Sugeno fuzzy for neutral differential systems with mixed time-varying delays] and the work in this paper.

**Author response:** Many thanks for your valuable suggestion. We have added in the **Remark 7** and add referent.

**Author action:**

**Remark 7** New results on robust exponential stability of Takagi - Sugeno fuzzy for neutral differential systems with mixed time-varying delays [40] only focus on exponential stability for neutral differential equations of the uncertain Takagi - Sugeno fuzzy system. This paper studies exponential stable and exponentially passive neutral differential equations of the uncertain Takagi - Sugeno fuzzy system for further improvement. Furthermore, we consider mixed interval time-varying delays: mixed interval discrete time-varying delay, interval distributed time-varying delay, and interval neutral time-varying delay, i.e., $\sigma(t)$ interval discrete time-varying delay which satisfies $0\leq\sigma_{1}\leq\sigma(t)\leq\sigma_{2}$, $h(t)$ is interval distributed time-varying delays which satisfy $0\leq h_{1}\leq h\left( t \right)\leq h_{2}.$ and $\tau(t)$ is interval neutral time-varying delay which satisfy $0\leq\tau_{1}\leq\tau(t)\leq\tau_{2}$.

[40] Tranthi J, Botmart T, Weera W, La-inchua T, Pinjai S. New results on robust exponential stability of Takagi - Sugeno fuzzy for neutral differential systems with mixed time-varying delays. Math Comput Simul. 2022; 201 : 714-738.

4. The novelty of this study has been explicitly stated.

**Author response:** We appreciate your compliments.

**Author action: -**

5. What is the research object in this paper?

**Author response:** this paper investigates the delay-dependent approach to exponential passive of T-S fuzzy for uncertain neutral dynamical system with discrete interval and distributed time-varying delays. Based on delay partitioning, a LKF is constructed to obtain several improved delay-dependent passivity conditions which guarantee the passivity of T-S fuzzy for uncertain neutral dynamical system. We consider the additional useful terms with the distributed delays and estimate some integral terms by Wirtinger’s inequality provided a tighter lower bound than Jensen’s inequality. The Lyapunov–Krasovskii functional contains single, double, triple, and quadruple integral terms, in which full information on the delays and a state variable is used. Therefore, the construction and the technique for computation of the Lyapunov–Krasovskii functional are the main keys to improving the results of this work. In the proof of Theorems 1 improved Wirtinger’s single integral inequality, a novel triple integral inequality, and convex combination technique are used to bound the derivative of Lyapunov– Krasovskii functional, which provide a tighter bound than the inequalities. All of these lead to a reduction of the conservatism of our results compared to those in some exciting works and, in particular, numerical examples. However, the complex computation of the Lyapunov–Krasovskii functional leads to the LMI derived in this work which contains much information about the system. Hence, for further work, it is interesting for researchers to improve the technique for a simple Lyapunov–Krasovskii functional and also achieve better results.

**Author action: -**

6. The format and English writing of the paper should be improved.

**Author response:** We really appreciate your suggestion. We have checked the grammatical problems already in this paper.

**Author action:** We have corrected the manuscript by rewriting it as follows:

1. On page 1, line 3 “The Newton-Leibniz theory” should be “the Newton-Leibniz theory”

2. On page 1, line 10 “Lastly, the examples of Numerical are presented the performance of our criteria with the results obtained summarize some of the previous results as well.” should be “Lastly, some numerical examples illustrate the performance of our criteria base on the results obtained and summarize some of the previous achievement as well.”

3. On page 3, line 95 “Fang Liu, et al. [?], Li et al. [?], Lien et al. [7, 28] and Pin-Lin Liu [?].” should be “by Fang Liu, et al. [26], Li et al. [27], Lien et al. [7, 28] and Pin-Lin Liu [29].”

4. On page 3, line 106 “that do not appear in [?,?,?, 7, 28, 30].” should be “that do not appear in [7, 26{30].”

5. On page 4, line 110 “previous literature [?,?,?, 7, 28, 30].” should be “previous literature [7, 26{30].”

6. On page 5, line 133 “Definition 1 [?] The system (1)” should be “Definition 1 [31] The system (1)”

7. On page 6, line 1 “Lemma 1 [?]” should be “Lemma 1 [32]”

8. On page 6, line 6 “Lemma 2 [?], [?]” should be “Lemma 2 [33], [34]”

9. On page 6, line 7 “the following inequality hold:” should be “the following inequality holds:”

10. On page 6, line 14 “Lemma 3 [?]” should be “Lemma 3 [35]”

11. On page 6, line 15 “the following inequality hold:” should be “the following inequality holds:”

12. On page 6, line 21 “Lemma 4 [?]” should be “Lemma 4 [36]”

13. On page 6, line 22 “the following inequality hold:” should be “the following inequality holds:”

14. On page 18, line 142 “The proof is completes” should be “The proof is completed”

15. On page 20, line 155 “exponential stability” should be “exponential stable”

7. The conclusion is descriptive. It is void of quantitative and numerical improvement and comparison.

**Author response:** We appreciate your valuable remark. We have already rewritten the conclusion part.

**Author action:** We updated the manuscript as follows:

This study rectifies the exponentially passive analysis of the neutral difference of the uncertain Takagi-Sukeno fuzzy system with neutral, discrete, and distributed interval time-varying delay. By spending the Newton-Leibniz formulas, Lyapunov-Krasovskii Functions (LKF), zero equations, and matrix inequality techniques. The form of linear matrix inequalities (LMIs) is constructed from the exponentially passive, in which the numerical efficiency can be verified. Hence, this study shows the example of numbers to demonstrate the effectiveness of our theoretical results and to illustrate the results we have obtained are less conservative than the results available in other works: according to Corollary 2, we get the upper bounds of the time-varying delay $\sigma_{2}$for various $\sigma_{d}$ and$\alpha$. Summarize them in Table 3 for comparison with the results obtained in [7, 26–29]. It is concluded that our results have the upper bounds of the time-varying delay $\sigma_{2}$ at the amount of 1.1121 for $\sigma_{d}$ = 0.9 and $\alpha$ = 0.9. Moreover, in Corollary 3, we get the upper bounds of the time-varying delay$\sigma_{2}$. Summarize them in Table 6 for comparison with the results obtained in [30]. It is concluded that our results have the upper bounds of the time-varying delay $\sigma_{2}$ at the amount of 0.5021 for $\tau$= 0.2450 and $\alpha$ = 0.23. We can see that our obtained results are less conservative than some existing results. In future work, the derived results and methods in this paper are expected to be applied to other systems such as fuzzy generalized complex-valued, guaranteed cost, pinning control of neural networks for impulsive effects on stability and passivity analysis, and so on [41-45].
